# Electronic Cigarette Perception in Baltimore High Schools

**DOI:** 10.31586/ojp.2025.6203

**Published:** 2025-10-27

**Authors:** Pardis Mohammad Zadeh, Rifath Ara Alam Barsha, Chidubem Egboluche, Payam Sheikhattari, Shervin Assari

**Affiliations:** 1 Department of Epidemiology and Biostatistics, School of Medicine, Kurdistan University of Medical Sciences, Sanandaj, Iran; 2 University of Mississippi Medical Center, Jackson, MS 39216, USA; 3 School of Community Health & Policy, Morgan State University, Baltimore, MD 21251, USA; 4 Department of Internal Medicine, Charles R. Drew University of Medicine and Science, Los Angeles, CA 90059, USA

**Keywords:** Social Determinants of Health, E-Cigarette Perception, Adolescents, Baltimore, Low-Income Youth, Black Youth, Vaping Perceptions, Parental Education, Parental Employment, Gender Differences, Grade Level, Age Differences, Cross-Sectional Survey, Health Disparities, Urban Youth

## Abstract

**Background:**

Electronic cigarette (e-cigarette) use among adolescents is a growing public health concern, particularly in low-income and Black communities. However, little is known about how social determinants of health shape e-cigarette perceptions in this population.

**Aims:**

This study examined social determinants associated with perceptions of e-cigarette safety among Baltimore high school students.

**Methods:**

A cross-sectional survey (CEASE Youth: School Survey) was conducted with 604 Baltimore high school students aged 14–20. Participants completed a questionnaire assessing perceptions of e-cigarette safety, as well as parental education, race/ethnicity, parental employment, household composition, and community tobacco use.

**Results:**

Higher parental education was associated with lower perceived e-cigarette safety among students. Students in higher grades also reported lower perceived e-cigarette safety. In contrast, male students—particularly those in upper grades—were more likely to perceive e-cigarettes as safe. Race/ethnicity, household composition, parental employment, and community tobacco exposure were not associated with perceived e-cigarette safety.

**Conclusion:**

Higher parental education, female gender, and being in higher grades were associated with perceiving e-cigarettes as unsafe. These findings highlight the need for targeted interventions to address vaping perceptions among youth in urban settings.

## 1. Introduction

Adolescents’ and young adults’ use of electronic cigarette (e-cigarette) have become a significant public health concern [1,2]. The rapid rise in popularity of youth e-cigarette and vaping is fueled by several factors, including widespread availability, aggressive marketing by manufacturers, and a pervasive perception that e-cigarettes are a safer alternative to traditional tobacco products like cigarettes or cigars [3,4]. However, mounting scientific evidence challenges this perception. It highlights significant health risks associated with e-cigarette use. These include nicotine addiction, respiratory harm, and potential long-term cardiovascular and neurological effects [5,6]. Despite these concerns, the favorable view of e-cigarettes among young people persists, contributing to increasing rates of use and posing challenges for public health interventions [7–9]. National surveys consistently report that large proportions of adolescents view e-cigarettes as relatively safe. For example, one study found that 43% of youth perceived e-cigarettes as less harmful than cigarettes [10]. In another national survey, with a total of 16845 final participants, about 2–5 % adolescents described e-cigarettes as posing no harm and about 30–48% believed e-cigarettes cause a lot of harm [11]. A different study reported that 16.6% of middle and high school students described occasional e-cigarette use as posing “no” or only “little” harm [12]. These misperceptions are concerning because they normalize favorable attitudes toward e-cigarette safety across diverse youth populations [13].

Social determinants, including race, parental education, parental employment status, household structure, and tobacco use in the community, are associated with adolescents’ perceptions of e-cigarette safety [14–16]. Race, particularly Black identity, might be linked to greater exposure to targeted e-cigarette marketing, which incorporates cultural cues and is associated with vaping behaviors [17]. Lower parental education levels are correlated with lower health literacy and protective behaviors, which may be associated with higher youth vaping rates through informed discussions about risks [18,19]. Parental unemployment or underemployment—prevalent in low-income communities—is associated with limited resources for health education. This may be linked to more permissive attitudes toward vaping. Single-parent households, compared to double-parent households, might be associated with lower supervision. This could correlate with reduced substance use rates among adolescents, including vaping uptake. Community tobacco use is associated with greater visibility of nicotine consumption, which may be linked to perceptions of e-cigarettes as socially acceptable and low-risk. These determinants collectively characterize the social environment in which youth navigate vaping-related decisions.

E-cigarette attitudes, such as e-cigarette perception, are pivotal psychological and cognitive factors associated with e-cigarette use among youth populations because they are linked to how youth evaluate the risks and benefits of vaping [4,20,21]. Pro-e-cigarette attitudes—such as perceiving e-cigarettes as safe, socially desirable, or a stress-reliever—are associated with higher rates of experimentation and regular use [22–26]. These attitudes are linked to greater exposure to health risks [23]. Examples include nicotine dependence and lung injury [6]. Perceptions of e-cigarette safety are particularly critical in vulnerable populations [4]. In these groups, socioeconomic challenges and limited access to health education may increase the appeal of vaping [27–29]. For instance, a study of urban Black adolescents in California found that many participants described e-cigarettes as safer than cigarettes, reflecting a widespread belief in reduced harm [30]. Nationally representative research also shows that adolescents from low-income households were significantly more likely to underestimate the health risks of e-cigarettes compared to peers from higher-income households [29]. A systematic review further concluded that minority and disadvantaged adolescents disproportionately perceive e-cigarettes as less harmful than cigarettes [14]. Vaping is often seen as a low-risk alternative to traditional cigarettes [10,30]. These findings suggest that favorable safety perceptions are not evenly distributed, but rather concentrated in communities facing socioeconomic disadvantage and targeted marketing exposure, underscoring the importance of examining e-cigarette perception in such contexts. Moreover, pro-e-cigarette attitudes might be shaped by a complex interplay of social, cultural, and environmental factors [22,25,26]. These include peer influences, family dynamics, and community norms [26]. Such factors are linked to the normalization of e-cigarette use [26,31]. Understanding attitudes such as e-cigarette perception is essential for designing targeted interventions, as misperceptions about e-cigarette perception and efficacy may be associated with use patterns that contribute to health disparities [13,29].

The Theory of Planned Behavior (TPB), developed by Icek Ajzen, highlights the role of attitudes as a key determinant of behavior. According to this theory, e-cigarette perception might be highly relevant to understanding e-cigarette use among low-income and Black high school students. Attitudes reflect an individual’s evaluation of vaping, such as perceiving e-cigarette as safe or trendy, which is common among youth exposed to targeted marketing [32]. Studies show that positive attitudes toward vaping, often reinforced by social media, predict stronger intentions to vape, which in turn predict actual use [33]. Males are more likely to view vaping as a status symbol. Females often perceive it as a stress-reliever. Older students report more favorable attitudes than younger peers [26,31,34]. TPB-guided interventions can target attitudes toward vaping. They use culturally tailored, gender- and age-specific education to address misperceptions of e-cigarette safety. This approach can effectively reduce vaping rates among low-income and predominantly Black high school students [32].

Gender and grade differences may exist in e-cigarette perception and vaping behaviors. For instance, males may report vaping in relation to social status. Females may report it in relation to stress coping. Higher-grade students exhibit stronger pro-vaping attitudes. This may be due to cumulative social exposures. Conversely, lower e-cigarette perception, associated with targeted health campaigns, are linked to lower vaping use [26,27,29,34]. For example, efforts by the Truth Initiative were associated with reduced youth vaping through greater awareness of harms [35,36].

### Aims

This study aimed to examine social determinants associated with e-cigarette perception among low-income and predominantly Black high school students in Baltimore. It assesses e-cigarette perception while exploring gender and grade variations in the associations between social determinants and e-cigarette safety perceptions. Baltimore’s population is predominantly low-income and Black. It faces unique social factors, including economic hardship, limited healthcare access, and systemic inequities. These may be associated with adolescents’ perceptions of e-cigarette safety. Baltimore’s high prevalence of youth vaping, coupled with its urban environment, makes it a critical setting for examining adolescents’ perceptions of e-cigarette safety and related social determinants. Findings from this research aim to inform the development of school-based prevention programs and evidence-based policies to address electronic cigarette use among vulnerable youth in similar urban settings.

## 2. Materials and Methods

### Design and Setting

This is a cross-sectional study conducted in Baltimore, Maryland, in 2024 and 2025. Data from the CEASE Youth [37]: School Survey, an online survey given to Baltimore City high school students, was used in this cross-sectional study. The survey was the result of a collaboration between the American Lung Association’s Not On Tobacco (N-O-T) campaign and Morgan State University’s CEASE program. This survey study was conducted at a few public high schools in Baltimore. It was intended to investigate tobacco use and associated behavioral and demographic characteristics. A self-completed, private online approach was used to collect data in order to encourage widespread participation and reduce response bias. The CEASE Youth Survey obtained informed consent before starting. It was administered via an electronic platform. The survey collected non-identifying information, such as the current date, the name of the participating school, a four-character code generated from the respondent’s phone number and initials, and the month and year of their birth. No personally identifying information was recorded to protect participant privacy. As a thank you present, each student who completed the survey received a $10 digital gift card.

### Population

The eligibility criteria for this research were: 1) attending a high school in Baltimore inner city in 2024 and 2025, and 2) provision of consent to participate. The target population comprised low-income high school students (grades 9–12) in Baltimore, with a focus on those identifying as Black. The final sample consisted of 604 students.

### Study Variables

The primary outcome variable, e-cigarette perception, was assessed using an ordinal scale ranging from 0 to 9. Lower scores indicate that participants perceived e-cigarettes as less safe than traditional cigarettes, whereas higher scores indicate that participants perceived e-cigarettes as safer than traditional cigarettes. This score was derived from responses to three targeted questions designed to capture a range of e-cigarette perception scores: (1) “Electronic cigarettes only contain water and flavored juice,” (2) “Electronic cigarettes are safer than regular cigarettes,” and (3) “Electronic cigarettes are less addictive than cigarettes.” Each question offered four response options—strongly agree, agree, disagree, and strongly disagree—with higher composite scores indicating greater e-cigarette perception, an undesirable outcome from a public health perspective. For the purposes of this analysis, the composite score was treated as a continuous variable to evaluate incremental differences in e-cigarette safety perceptions.

Independent variables included gender, age, grade, race, parental education, household income, and community tobacco use. Parental education level was measured on an ordinal scale. Current employment status was dichotomized into unemployed or employed. Household composition was classified as single-parent or double-parent. Community tobacco use was assessed on an ordinal scale from None to All, based on response to the question “Have you seen people around your community using tobacco products”?

### Statistical Analysis

Descriptive statistics were calculated to summarize the sample and variable distributions. Bivariate analyses employed independent-samples t-tests with equal variances to compare mean e-cigarette perception scores between binary groups and one-way analysis of variance (ANOVA) for multi-categorical variables. Bartlett’s test validated the equal variance assumption (all p > 0.05). Multivariate analyses utilized multiple linear regression to assess the combined effects of the aforementioned predictors on the outcome. *Model 1* displays the results of the independent variable, while *Models 2 and 3* illustrate stratified models based on age and gender. Results are shown as “b” values with 95% confidence intervals (CIs). All statistical analyses were performed using Stata version 16.0 (StataCorp LLC, College Station, TX).

### Institutional Review Board (IRB)

Youth participants in the study provided assent, and adult parents provided informed consent before beginning the survey and approved their children’s participation. The study was approved by the Morgan State University Institutional Review Board.

## 3. Results

### Descriptive Data

The study sample included 604 high school students aged 14–20 years from Baltimore. The overall mean e-cigarette perception score was 2.72 (95% CI: 2.57, 2.87), indicating a neutral-to-favorable perception of e-cigarette safety across the participants, meaning that many participants perceived e-cigarettes relatively safe compared to traditional cigarettes. Students 16 years and under exhibited a mean score of 2.79, marginally higher than the 2.63 for upper-grade students. Gender differences were subtle, with females scoring 2.64 (95% CI: 2.43, 2.84, n = 326, 55.6%) and males 2.85 (95% CI: 2.61, 3.09, n = 260, 44.4%), indicating a slight difference in e-cigarette perception between genders. Racial composition showed 533 Black or African American students (88.2%; mean = 2.72, 95% CI: 2.56, 2.88), 20 White students (3.3%; mean = 2.65, 95% CI: 1.97, 3.33), and 51 students of other race / ethnicity (8.4%; mean = 2.78, 95% CI: 2.19, 3.37), with little variation across groups. Parental education level, ranging from lower to higher, displayed a notable trend in e-cigarette perception score mean = 2.96, 2.86, 2.67, 2.60, 2.46, respectively. Parental employment status revealed that students with unemployed parents (n = 87, 14.4%) had a mean e-cigarette perception score of 2.62 (95% CI: 2.10, 3.14), slightly lower than those with employed parents (n = 515, 85.3%; mean = 2.74, 95% CI: 2.58, 2.90), suggesting a modest difference in perceptions. Household composition showed that students from single-parent households (n = 378, 62.6%) reported a mean e-cigarette perception score of 2.69 (95% CI: 2.50, 2.88), compared to 2.78 (95% CI: 2.51, 3.05) for those from double-parent households (n = 225, 37.3%), indicating negligible variation. Community tobacco use displayed a non-linear trend, with no clear association with e-cigarette perception scores. Table 1 provides a detailed summary of descriptive statistics stratified by key social determinants.

Table 2 presents the overall linear regression model analysis for social determinants of e-cigarette perception. Regarding grade level, 10th and 12th grades (b = −0.44, 95% CI: −0.93, 0.04, and b = −0.43, 95% CI: −0.89, 0.03, respectively) showed a trend toward lower e-cigarette perception compared to 9th grade, suggesting that higher-grade levels may be associated with reduced e-cigarette safety perceptions, though not statistically significant. For gender, males showed no significant difference in e-cigarette perception compared to females (b = 0.33, 95% CI: −0.01, 0.67). Racial comparisons revealed no significant differences, indicating that racial background was not associated with variations in e-cigarette perception. Parental education levels showed a progressive trend, with the highest level significantly associated with lower e-cigarette perception compared to the lowest level (b = −0.70, 95% CI: −1.35, −0.05); students with highly educated parents reported lower e-cigarette perception. Current employment status showed no significant association. Household composition indicated no association, with double-parent households showing no difference from single-parent households. Community tobacco use levels showed no significant associations with e-cigarette perception.

For both age groups, under 16 and older, the multivariate model shows 12th grade trended toward lower e-cigarette perception, though not statistically significant. In the older group, male gender was significantly associated with higher e-cigarette perception compared to females (b = 0.86, 95% CI: 0.29 to 1.42), indicating that males in this group reported higher e-cigarette perception. In contrast, older male students showed no association, suggesting that gender was not associated with differences in perceptions in this group. In both groups, racial comparisons showed no significant associations, indicating that race was not associated with e-cigarette perception. In the younger group, parental education levels indicated a trend, with the highest level associated with lower e-cigarette perception (b = −0.91 95% CI: −1.75, −0.07), suggesting that higher parental education was associated with higher e-cigarette perception among younger students. Parental employment status, household composition (single- vs. double-parent), and community tobacco use showed no significant associations with e-cigarette perception in either age group (Table 3).

Table 4 shows that in the gender-stratified linear model, 12th grade was significantly associated with lower e-cigarette perception in females (b = −0.80, 95% CI: −1.39 to −0.22), indicating that females in their final year reported lower e-cigarette perception. Racial comparisons showed no significant associations with e-cigarette perception in either gender. In the female group, higher parental education, particularly at the graduate degree level, was significantly associated with lower e-cigarette perception (b = −0.83, 95% CI: −1.63 to −0.02), indicating that females with more educated parents perceived e-cigarettes as less safe. In contrast, parental education showed no association with e-cigarette safety perceptions in male students. Other social determinants, including current employment status, household status, and community tobacco use, showed no significant associations with e-cigarette perception among either gender.

## 4. Discussion

This cross-sectional study of 604 predominantly low-income Black high school students in Baltimore offers important insights into how social determinants shape perceptions of e-cigarette safety—a key factor influencing youth vaping behavior. On average, students reported neutral-to-favorable views of e-cigarette safety, suggesting that many adolescents considered e-cigarettes relatively safe compared to traditional cigarettes. Parental education showed a consistent association: students whose parents had higher education levels reported lower e-cigarette perception. Grade level also mattered, with 12th graders generally showing lower perceptions of e-cigarette safety than students in earlier grades. In contrast, race, parental employment, household composition, and community tobacco use were not associated with safety perceptions. Gender and grade appeared to interact with these patterns. Male students in higher grades tended to report greater e-cigarette perception, whereas female students in 12th grade reported lower perceived safety. Furthermore, the protective effect of higher parental education against favorable e-cigarette safety perceptions was stronger among students in lower grades and among females.

Research on overall e-cigarette safety perceptions in low-income and minority youth notes neutral or more favorable e-cigarette safety perceptions, aligning with our neutral mean score [29], though some urban studies differ [38,39]. Studies show higher parental education is associated with lower e-cigarette perception [29,40], consistent with our data. Studies report lower e-cigarette safety perceptions among higher-grade adolescents, consistent with our trend of reduced perceptions in upper-grades, particularly 12th grade [41]. Regarding gender, research indicates lower e-cigarette safety perceptions among females. This aligns with our findings for 12th-grade females. Higher perceptions among males are consistent with our results for higher-grade males [13,29]. Prior studies frequently report racial differences in e-cigarette safety perceptions, with Black adolescents often showed higher e-cigarette perception, contrasting with our finding of no racial differences in e-cigarette perception [30].

The observed associations likely stem from a complex interplay of health literacy, social norms, peer influences, and systemic inequities prevalent in urban low-income settings [41,42]. Higher parental education is associated with informed family discussions about the health risks of vaping, which may be linked to more cautious perceptions of e-cigarette safety among youth, particularly younger students (≤16 years) and females [43]. This association may be stronger among lower-grade students due to greater parental influence during early adolescence and among females due to heightened responsiveness to health-focused communication within family structures [43,44]. Gender differences are associated with distinct social factors. Males, especially higher-grade males, may perceive e-cigarettes as safer, viewing them as socially desirable or status-enhancing. This may be linked to targeted e-cigarette marketing in minority communities [45]. In contrast, females, particularly in 12th grade, may report lower e-cigarette safety perceptions. This could be associated with exposure to health-focused messaging through school programs, social networks, or community health initiatives [46]. The lack of association between community tobacco use and e-cigarette perception may be linked to widespread e-cigarette advertising. This includes social media, television, and online platforms [47]. Such advertising may have a stronger correlation with students’ perceptions than exposure to traditional tobacco use in family, community, or school environments [48]. This is particularly evident in urban low-income settings, where access to accurate health education is often limited. In such contexts, pervasive marketing may be associated with the normalization of e-cigarette use. It may overshadow local tobacco use norms. This marketing correlates with e-cigarette safety perceptions, particularly among males exposed to culturally targeted advertisements [16,49].

The results potentially have some implications for public health in low-income, predominantly Black urban communities. The association between parental education and health literacy highlights the potential value of community-based programs to support parents with lower education levels in guiding youth away from vaping [50]. Gender-specific interventions are essential. Campaigns targeting higher-grade males should highlight long-term health risks to counter social desirability biases. For females, interventions should reinforce cautious perceptions of e-cigarette safety through tailored education [51,52]. Policy measures, such as restricting e-cigarette marketing in minority communities, could address systemic drivers of neutral e-cigarette safety perceptions, reducing vaping uptake and health disparities [4]. School-based programs grounded in frameworks like the Theory of Planned Behavior could reshape perceptions of e-cigarette safety, preventing vaping initiation in vulnerable populations [53].

### 4.1. Future Research

Future studies should employ longitudinal designs to better clarify potential causal pathways between social determinants and e-cigarette perception, with particular attention to how parental education shapes these perceptions over time. Intersectional analyses that consider race, income, and gender may uncover more nuanced drivers of vaping attitudes. Evaluating culturally tailored interventions—such as anti-vaping curricula and parental engagement programs—in urban school settings could inform scalable prevention strategies. In addition, examining the influence of digital media exposure on e-cigarette safety perceptions may provide insight into broader systemic factors shaping youth beliefs.

### 4.2. Limitations

This study has several limitations. Its cross-sectional design precludes causal inferences. Reliance on self-reported data may introduce response bias. The sample, drawn from Baltimore high school students, may not generalize to other regions or to non-school-attending youth. Treating e-cigarette perception as a continuous variable may oversimplify underlying ordinal responses. Furthermore, unmeasured influences, such as targeted marketing exposures, could affect student perceptions.

## 5. Conclusions

This study suggests that higher parental education and female gender in upper grades are associated with lower e-cigarette perception among low-income Black youth in Baltimore, while race and community tobacco use showed little association. These findings highlight the importance of targeted prevention efforts. Interventions that address parental education and incorporate gender-sensitive messaging may help reduce misperceptions of e-cigarette safety and lower vaping prevalence. Public health strategies should also address broader systemic inequities through policy and community engagement, with the goal of reducing e-cigarette–related harms in vulnerable populations and promoting more equitable health outcomes. Such approaches would align with national priorities to curb youth e-cigarette use.

## Figures and Tables

**Table 1.** Descriptive Statistics of Social Determinants and E-cigarette perception Among Baltimore High School Students.

Variables	N (%)	E-cigarette perception Score
		Mean (95% CI)
Total	604 (100)	2.72 (2.57, 2.87)
Age		
Less than or equal 16	360 (59.60)	2.79 (2.59, 2.98)
More than 16	244 (40.40)	2.63 (2.38, 2.88)
Gender		
Female	326 (53.97)	2.64 (2.43, 2.84)
Male	260 (43.05)	2.85 (2.61, 3.09)
Race		
Black	533 (88.25)	2.72 (2.55, 2.88)
White	20 (3.31)	2.65 (2.04, 3.26)
Other	51 (8.44)	2.78 (2.25, 3.31)
Grade		
9^th^	143 (23.68)	3.00 (2.68, 3.33)
10^th^	137 (22.68)	2.60 (2.28, 2.92)
11^th^	137 (22.68)	2.71 (2.41, 3.00)
12^th^	180 (29.80)	2.63 (2.33, 2.92)
Grade Point Average		
F and D	22 (3.64)	3.23 (2.53, 3.92)
C	149 (24.67)	3.06 (2.74, 3.37)
B	272 (45.03)	2.72 (2.50, 2.94)
A	161 (26.66)	2.34 (2.04, 2.65)
Parental Education		
Less than High School Diploma	75 (12.42)	2.96 (2.56, 3.36)
High school Graduation	215 (35.60)	2.85 (2.59, 3.12)
Some College	129 (21.36)	2.67 (2.33, 3.00)
College Graduation	78 (12.91)	2.60 (2.19, 3.01)
Graduate Degree or Higher	104 (17.22)	2.46 (2.10, 2.82)
Parental Employment		
Unemployed	87 (14.40)	2.62 (2.10, 3.14)
Employed	515 (85.26)	2.74 (2.58, 2.90)
Household Status		
Single-Parent	378 (62.58)	2.69 (2.50, 2.88)
Double-Parent	225 (37.25)	2.78 (2.51, 3.05)
Community Tobacco Use		
None	30 (4.97)	2.43 (1.57, 3.29)
A few	103 (17.05)	3.03 (2.65, 3.41)
Some	142 (23.51)	2.39 (2.10, 2.67)
Most	186 (30.79)	2.71 (2.45, 2.98)
All	73 (12.09)	2.73 (2.29, 3.16)

**Table 2.** Linear Regression Analysis of Associations Between Social Determinants and E-cigarette perception Among Baltimore High School Students

	b (95% CI)
Gender	
Female	Ref.
Male	0.33 (−0.01, 0.67)
Race	
White	Ref.
Black	−0.02 (−0.89, 0.85)
Other	−0.22 (−1.25, 0.80)
Grade	
9^th^	Ref.
10^th^	−0.44 (−0.93, 0.04)
11^th^	−0.24 (−0.72, 0.25)
12^th^	−0.43 (−0.89, 0.03)
Grade Point Average	
F and D	Ref.
C	−0.26 (−1.18, 0.65)
B	−0.67 (−1.57, 0.22)
A	−0.95 (−1.86, −0.03) *
Parental Education	
Less than High School Diploma	Ref.
High School Graduation	−0.37 (−0.93, 0.20)
Some College	−0.41 (−1.03, 0.20)
College Graduation	−0.40 (−1.09, 0.29)
Graduate Degree or Higher	−0.70 (−1.35, −0.05) *
Parental Employment	
Unemployed	Ref.
Employed	0.31 (−0.18, 0.80)
Household Status	
Single-Parent	Ref.
Double-Parent	0.20 (−0.15, 0.55)
Community Tobacco Use	
None	Ref.
A Few	0.59 (−0.20, 1.39)
Some	−0.05 (−0.82, 0.71)
Most	0.30 (−0.44, 1.05)
All	0.20 (−0.63, 1.04)

Note:

* *p* < 0.05

**Table 3.** Linear Regression Analysis of Social Determinants Associated with E-cigarette perception by Age Among Baltimore High School Students

	Less than or equal 16	Older than 16
	b (95% CI)	b (95% CI)
Gender		
Female	Ref.	Ref.
Male	0.03 (−0.42, 0.47)	0.86 (0.29, 1.42) *
Race		
White	Ref.	Ref.
Black	−0.31 (−1.53, 0.92)	0.60 (−0.73, 1.93)
Other	−0.36 (−1.78, 1.05)	0.19 (−1.44, 1.82)
Grade		
9^th^	Ref.	Ref.
10^th^	−0.48 (−0.98, 0.02)	0.39 (−2.99, 3.76)
11^th^	−0.30 (−0.87, 0.26)	−0.40 (−3.16, 2.35)
12^th^	−0.57 (−4.26, 3.12)	−0.62 (−3.35, 2.11)
Grade Point Average		
F and D	Ref.	Ref.
C	−1.09 (−2.43, 0.26)	0.94 (−0.44, 2.33)
B	−1.22 (−2.55, 0.10)	0.29 (−1.07, 1.66)
A	−1.42 (−2.77, −0.07) *	−0.19 (−1.57, 1.18)
Parental Education		
Less than High School Diploma	Ref.	Ref.
High School Graduation	−0.64 (−1.38, 0.10)	−0.05 (−0.95, 0.86)
Some College	−0.66 (−1.46, 0.13)	−0.01 (−1.03, 1.01)
College Graduation	−0.42 (−1.34, 0.51)	−0.53 (−1.62, 0.56)
Graduate Degree or Higher	−0.91 (−1.75, −0.07) *	−0.47 (−1.53, 0.60)
Parental Employment		
Unemployed	Ref.	Ref.
Employed	0.24 (−0.46, 0.94)	0.25 (−0.46, 0.97)
Household Status		
Single-Parent	Ref.	Ref.
Double-Parent	0.30 (−0.15, 0.76)	−0.03 (−0.63 0.56)
Community Tobacco Use		
None	Ref.	Ref.
Few	0.52 (−0.61, 1.66)	0.73 (−0.46, 1.91)
Some	−0.13 (−1.23, 0.97)	0.24 (−0.88, 1.35)
Most	0.24 (−0.85, 1.33)	0.40 (−0.67, 1.47)
All	0.03 (−1.15, 1.21)	0.60 (−0.68, 1.88)

Note:

* *p* < 0.05

**Table 4.** Linear Regression Analysis of Social Determinants Associated with E-cigarette perception by Gender Among Baltimore High School Students

	Female	Male
	b (95% CI)	b (95% CI)
Race		
White	Ref.	Ref.
Black or African American	−0.65 (−1.77, 0.48)	0.94 (−0.48, 2.36)
Other	−0.54 (−1.89, 0.81)	0.14 (−1.51, 1.80)
Grade		
9^th^	Ref.	Ref.
10^th^	−0.39 (−1.00, 0.22)	−0.59 (−1.41, 0.24)
11^th^	−0.39 (−1.01, 0.23)	0.00 (−0.82, 0.82)
12^th^	−0.80 (−1.39, −0.22) *	0.06 (−0.71, 0.84)
Grade Point Average		
F and D	Ref.	Ref.
C	−0.21 (−1.31, 0.89)	−0.09 (−1.75, 1.57)
B	−0.54 (−1.62, 0.55)	−0.56 (−2.20, 1.07)
A	−0.93 (−2.02, 0.16)	−0.71 (−2.40, 0.98)
Parental Education		
Less than High School Diploma	Ref.	Ref.
High School Graduation	−0.17 (−0.89, 0.54)	−0.75 (−1.73, 0.23)
Some College	−0.34 (−1.11, 0.42)	−0.69 (−1.77, 0.39)
College Graduation	−0.22 (−1.12, 0.69)	−0.92 (−2.09, 0.25)
Graduate Degree or Higher	−0.83 (−1.63, −0.02) *	−0.68 (−1.80, 0.45)
Parental Employment		
Unemployed	Ref.	Ref.
Employed	0.16 (−0.47, 0.79)	0.35 (−0.45, 1.16)
Household Status		
Single-Parent	Ref.	Ref.
Double-Parent	0.02 (−0.47, 0.51)	0.39 (−0.15, 0.92)
Community Tobacco Use		
None	Ref.	Ref.
Few	0.47 (−0.82, 1.76)	0.41 (−0.70, 1.52)
Some	−0.53 (−1.81, 0.75)	0.30 (−0.70, 1.31)
Most	−0.01 (−1.26, 1.23)	0.51 (−0.47, 1.50)
All	−0.24 (−1.57, 1.08)	0.62 (−0.56, 1.80)

Note:

* *p* < 0.05

## Data Availability

Data will be available upon request.

## Abbreviations

The following abbreviations are used in this manuscript:

CI: Confidence Interval
CEASE: Communities Engaged and Advocating for Smoke-free Environments
N-O-T: Not On Tobacco
IRB: Institutional Review Board
TPB: Theory of Planned Behavior
ANOVA: Analysis of Variance
Ref.: Reference Category
b: Unstandardized Regression Coefficient
